# Community Health Scholars: a summer program developing a public health workforce pipeline for diverse high school students

**DOI:** 10.3389/fpubh.2023.1256603

**Published:** 2023-12-22

**Authors:** Michelle A. Tagorda-Kama, Denise C. Nelson-Hurwitz

**Affiliations:** Office of Public Health Studies, Thompson School of Social Work and Public Health, University of Hawai'i at Mānoa, Honolulu, HI, United States

**Keywords:** public health education, undergraduate public health, workforce, curriculum, outreach, recruitment, diversity

## Abstract

In response to the growing interest in public health and needs to both increase and diversify the public health workforce, opportunities to engage students early in their educational journey are essential. The University of Hawai'i at Mānoa launched the Community Health Scholars Program to provide activities for high school students to learn about and build enthusiasm for the field of public health. During the 6-week, in-person summer program, students underrepresented in higher education and who are from historically underrepresented communities completed a college course and participated in activities to enhance their successful entry into a higher education institution. The Community Health Scholars completed an introduction to public health course and gained an array of public health skills through different hands-on activities. The students gained self-confidence and expanded their social capital by attending workshops led by campus faculty, staff, and community partners. A final project highlighting what students learned about themselves and their community was part of a final program showcase. Here we share information about the process of developing the program, the components of the program curriculum, and feedback from both students of the initial cohort and program faculty, where overall satisfaction with the program was reported.

## 1 Introduction

Diversifying the public health workforce is essential in addressing the health issues facing our communities (1, 2). With increasingly diverse populations facing complex health issues, a public health workforce that not only represents diverse populations is urgently needed. While some countries face workforce challenges associated with migration and “brain-drain” (3, 4), in the United States, additional need exists to actively work toward addressing systemic issues to achieve health equity. One strategy to diversify the workforce is to diversify the educational pipeline to and through higher education (3, 4).

Diversifying the educational pipeline requires addressing the educational barriers that impact underrepresented and marginalized populations in higher education (5–8). Integrative approaches include providing affordable college credits, and college preparation activities to high school students which help to demystify the college-going experience for underrepresented and marginalized students (5, 9).

Recruitment into public health professions additionally face discipline-specific challenges. Barriers are in place that limit student interest in science, technology, engineering, and mathematics (STEM) education, in primary and secondary educational levels in the United States (10), in addition to barriers related to determinants of health that limit the ability of public health programming to accessing diverse communities (11). Even among high school STEM students, there is limited interest in life and social science, suggesting a need to diversify awareness and conversations of STEM pathways (10, 11).

The Association of Schools and Programs of Public Health (ASPPH) Framing the Future of Public Health Education reports the importance of expanding public health education and engagement in primary and secondary school settings (12). When colleges and universities provide high school students with the opportunity to explore public health careers and gain fundamental public health knowledge and skills, students can widen their perspectives of health (4). Engaging students early can also challenge them to think about the role they can play in making an impact in their communities from a public health perspective.

Facilitating meaningful relationships among a cohort of high school students and recent high school graduates creates space for shared learning and peer support during times of exploration and transition. Underrepresented and marginalized populations in higher education include ethnically diverse (e.g., Black and Indigenous People of Color), low-income, and first-generation college students who may feel uneasy about their college prospects. Summer learning can help students gain confidence and feel more prepared to face the next steps of their educational journey.

## 2 Pedagogical framework

High school students start to think about careers during their sophomore and junior years (13). Introducing this age demographic of students to public health provides an opportunity to expand their understanding of health professions beyond careers that are more familiar to them. Alongside career exploration is also the preparation needed to support students' readiness for higher education (13). By targeting rising high school seniors, students are developmentally primed to gain the confidence and skills to make it through what can be a stressful time in their lives (14). Partnerships with high school and college counselors, health pathway, and career and technical education teachers are essential to recruit students in this age group (15, 16) and demographic (17).

Summer learning programs provide students with an opportunity to explore, gain new skills, and develop more self-awareness. Summer programs with similar goals in recruitment and retention of students in higher education, such as GEAR UP (Gaining Early Awareness and Readiness for Undergraduate Programs), have reported strong outcomes in meeting these objectives as well as evidence supporting the need for additional support in the transition from high school to higher education (13, 14, 18). The availability and flexibility of summer opportunities also give students a chance to feel out what being a college student is like in a supportive environment. College faculty and staff are also generally more available during the summer months. Connecting students to campus resources during this time can help to build a sense of community and belonging for them even before they start college (13, 14).

Including ‘*ohana* (family) promotes a shared partnership for the student's success. Navigating college can be difficult for students and family members, especially for first-generation college-going students. ‘*Ohana* activities are not limited to immediate family members, but rather, are open to any participants a student identifies as a support person (e.g., neighbors, extended family members, or other supportive adults). These activities also give families and other members of students' social support system a chance to be part of the college-going experience, which helps students feel supported as well (19, 20).

In order to successfully engage with young learners, the curriculum was designed to be interactive and problem-based to promote authentic learning (21, 22). Activities that students complete involve connecting what they learned to their lived experiences, the campus community, and the communities they are from. Applying knowledge gained in public health to their personal lives allows students to think about ways they can improve their own health, their families' health, and more broadly, the health of their communities (21).

This is especially important for students from underrepresented and marginalized communities where there is an increasing need to train a diverse population of public health populations to reflect the communities they are from and identify with (3, 23, 24). Place-based learning strategies are essential for our students, especially in Hawai'i where local culture and indigenous roots place high significance on engagement with ‘*aina* (land) (25, 26). The linkages to local communities help students understand the need to learn about and address health disparities impacting the people of Hawai'i (19, 20). In applying place-based learning through engaging directly with ‘*aina* (land) students also gain outdoor exposure, which is associated with improved mental health, particularly stress management, among youth and adolescent populations (27, 28).

A culminating project where students share with their community what the needs, strengths, and opportunities are in their community builds confidence in communicating public health data, and ways to promote health. *Hō‘ike*, an end-of-program showcase translated from Hawaiian as an “exhibition, demonstration of knowledge” (29), celebrates students' work during the program and is a chance to share their summer learning experiences with their families and communities through a public event held on-campus. In addition to family members and other student-invited support people, faculty, staff, and University administrators are invited as well as local community partners (e.g., local policymakers, representatives from the Hawai'i Department of Health, high school teachers/administrators, and local non-governmental organizations), allowing for further opportunities for students to connect with community and professional partners.

## 3 Learning environment, learning objectives, and learning format

### 3.1 Learning environment

Summer program activities were conducted through the Office of Public Health Studies (OPHS) at the University of Hawai'i at Mānoa. Within the Thompson School of Social Work and Public Health, OPHS is a CEPH-accredited program of public health offering a bachelor of arts degree in public health, a minor in public health, as well as a Masters of Public Health (MPH) degree in four specializations, a Master of Science (MS) degree in epidemiology, and PhD degrees in both epidemiology and public health, focused on community-based participatory research.

The University of Hawai'i at Mānoa is a public, research-intensive university and the flagship campus for the University of Hawai'i system. Campus enrollment is 19,074 students, including 74.4% undergraduates (30). The University of Hawai'i system has self-identified as an indigenous-serving institution and a Hawaiian place of learning. This identity reflects the prioritization of indigenous populations and services within the administration and across multiple campuses.

Information about the program was disseminated among the OPHS faculty and staff to share with their respective contacts. Targeted recruitment activities included presentations to high school health courses and participation at college and career fairs held at various high schools. Broader range outreach was done via high school college counselors, health pathway, and career and technical education teachers. Local non-profit agencies (Hawai'i Public Health Association, Hawai'i Public Health Institute, and Hawai'i Youth Services Network) were asked to also share this opportunity with their respective email lists.

### 3.2 Learning objectives

Inspired by past and existing programs including Area Health Education Centers (AHEC) (31, 32), Health Careers Opportunity Programs (33), and a local post-baccalaureate program, ‘Imi Ho‘ola (34, 35), the Community Health Scholars Program was developed to meet six objectives.

- LEARN: Scholars complete PH 201—Introduction to Public Health alongside current college students.
- ENGAGE: Scholars engage in interactive public health activities to supplement knowledge gained in the PH 201 course.
- GROW: Scholars engage in personal and professional development.
- PREPARE: Scholars assess, then build, college readiness skills.
- ‘OHANA (Family): Participants have the opportunity to bring their family members to engage with events and workshops at specified intervals throughout the program to build familial support for college attendance and public health career pathways.
- COMMUNITY: Participants build connections with peers, near-peer college students, public health professionals, and members of the community.

### 3.3 Learning format

The 6-week Community Health Scholars Program was launched in the summer of 2022. It was in-session, in-person from mid-June through the near-end of July to accommodate the summer availability of high school students. The program was held daily on Mondays through Fridays from 9 am to 2:30 pm. The program was anchored by a morning college course attended by both scholar participants as well as college students looking to enroll in summer coursework. The course was offered from 9 am to 10:15 am, with two additional scholar program-specific sessions held for 90 min each from 10:30 am to 2:30 pm, inclusive of an hour-long lunch break. In general, Monday and Friday's sessions focused on student development, including college readiness and activities promoting self-reflection or social-emotional learning. Tuesday and Thursday sessions focused on public health content engagement activities, and Wednesday sessions focused on components of a scaffolded summer program community capstone project. An overview schedule is provided in Table 1.

**Table 1:** General overview schedule of summer scholars program.

	**Monday**	**Tuesday**	**Wednesday**	**Thursday**	**Friday**
9:00–10:15 am	PH 201: Introduction to Public Health Class				
10:15–10:30 am	Break				
10:30 am−12:00 pm	Student Development Activity	Public Health Engagement Activity	Summer Program Community Activity	Public Health Engagement Activity	Student Development Activity
12:00–1:00 pm	Lunch (Provided) + Occasional Community Visitors				
1:00 pm−2:30 pm	Student Development Activity	Public Health Engagement Activity/Movie	Summer Program Community Activity	Public Health Engagement Activity/Movie	Student Development Activity

#### 3.3.1 Foundational coursework

Throughout the program, a 3-credit introductory public health overview course was used as both the academic foundation for the program and as an important touchpoint for scaffolding public health content. The course itself includes such course objectives as identifying and discussing a range of real-world public health problems, identifying gaps in knowledge related to a public health problem, discussing ethical concerns and promoting ethical decision-making behaviors, engaging in self-directed inquiry and intellectual curiosity, and fostering cultural awareness and social justice. Class sessions include lectures, large and small group discussions, and in-class activities. Student scholars participating in the program enrolled in a course session offered simultaneously with current college students, resulting in both formal and informal opportunities for students to engage in a course alongside near-peers with similar interests, especially during small group discussions and in-class activities. Over 6 weeks, content themes discussed during class were applied during program-specific public health engagement activities. These activities served to reinforce academic content introduced during the course, as well as to engage students more actively in public health practice. Students earned three college credits (regularly priced at 1,500 USD), and a grade after completion of the course.

#### 3.3.2 Content engagement activities

Sample engagement activities include a mask assessment lab linked in epidemiology and public health biology, a state-level budget balancing scenario linked to health policy, the development of a video-based public service announcement to promote COVID-19 protection measures linked to behavioral health and communication, and a public health heroes activity linked to public health history.

The mask assessment was based on existing activities (36, 37) adapted to this program. Students worked in pairs and rotated through four different lab stations. Stations included a light test station, where students visually assess mask thickness using a flashlight, a respiration test station, where students attempt to blow out a candle from a standard distance while wearing each mask, a “sneeze” test station, where students use a spray bottle to assess the penetration of sprayed water, and finally, the physical layers test station, where students cut each mask to identify the number of physical layers. At each station, the student pairs conducted the associated lab test and rated the quality of each mask on a 5-point scale (one being the lowest quality and five being the highest). Three different masks were assessed—a cloth mask, a surgical mask, and a KN-95 mask. Findings were reported back to the larger group for broader discussion related to respiratory disease transmission and linkages to epidemiology.

The budget-balancing activity engaged students in communication and advocacy as small groups (3–4 per group) of students reviewed a mock state budget of roughly 50 line items accompanied by past funding requested. Students were then provided with mock state revenue projections and, assuming they were representing their home communities, asked to debate which line items to fund and at what level, based on past and requested funding information, in order to achieve a balanced budget. In a subsequent round of discussion, the small groups of students were asked to review their budget line items as a full class and debate the development of a final consensus budget. This activity allowed students to better understand health policy, gain a perspective of the policy-making process, and promote both communication and collaboration skills.

When discussing behavior change and health communication skills, students engaged in the development and production of a brief video-based public service announcement to promote COVID-19 protection measures among a peer group. Following lectures and discussions regarding behavior change theories and health communication, students worked in small groups to complete a worksheet to apply social marketing strategies. The worksheet was developed and modified by a faculty colleague at OPHS based on student need for a step-wise approach to brainstorming a theory-based behavioral intervention. Applying the worksheet, students were guided in developing a health message, then a messaging strategy, to promote COVID-19 protection measures among a peer group using a TikTok video format. Students recorded the videos and shared the final products, along with the associated worksheets.

Documentaries or public health-related fictional films and debriefing, instructor-led discussions were also incorporated into content-related programming. Using these films allowed students to see complex public health issues in action and on the ground, frequently as told from personal accounts centered on lived experiences, and forge connections between public health action and both the individuals and communities involved. Films shared included “I am Greta” (38), a documentary focused on the climate-change-related activism of Greta Thunberg, “Aftershock” (39), which discussed maternal mortality among black women in the U.S., and “*Ola*: Health is Everything” (40), a documentary highlighting social factors impacting health among populations in Hawai'i and showcasing promising practices to address these.

#### 3.3.3 Student development activities

Throughout the program, students were also involved in activities to prepare them for transitions to college, and in activities intended to promote social-emotional development. College readiness activities included guided activities and discussions where students identified their motivations in seeking a college education, discussed the college application process with an invited guest speaker from the University of Hawai'i at Mānoa Admissions Office, then developed college application materials.

Students also engaged directly with topics of particular relevance to underrepresented and first-generation college students. Program faculty were intentional in the inclusion of both discussions and activities centered on topics of financial literacy, budgeting, applying for scholarships, student success strategies (e.g., time management and note-taking strategies), and recognition of privilege. Academic partners and community experts were invited to share and discuss many of these topics with students. For example, a speaker from a local bank was invited to discuss financial literacy, and student support colleagues across campus were asked to share more about student success strategies.

Personal development activities included personal skill assessments such as the True Colors personality test (41, 42). Students also engaged in self-discovery activities intended to facilitate student identification of their motivations to pursue higher education based on the work of Sinek (43), and an activity of naming, describing, and then grouping emotions based on a resource by Brown (44).

#### 3.3.4 ‘*Ohana* (family) engagement activities

Engagement of family members was an integral component of the Community Health Scholars Program. Students were encouraged to invite family members to events and/or activities held every 2 weeks throughout the 6-week program. Both students and family members were invited to a brief virtual orientation held a week prior to the beginning of the program, so everyone would learn about what to expect and how to prepare for program participation, as well as have the opportunity to ask questions. During the program, ‘*ohana* activities were held for roughly 90 min on alternating Saturday mornings. These activities integrated with public health content and concepts students were engaged in within the program. Activities were also intended to promote community engagement and connection. A second ‘*ohana* activity intended to promote environmental community connections. A third activity focused on community advocacy and health communication strategies associated with advocacy and was supported by a faculty colleague with expertise in this area. The program celebration held the Saturday after the program's final day was the fourth, and last, ‘*ohana* event, highlighting the students, their work products of the semester, and sharing a journey of their program experiences.

#### 3.3.5 Final project and *Hō‘ike* (showcase)

**Figure 1:**
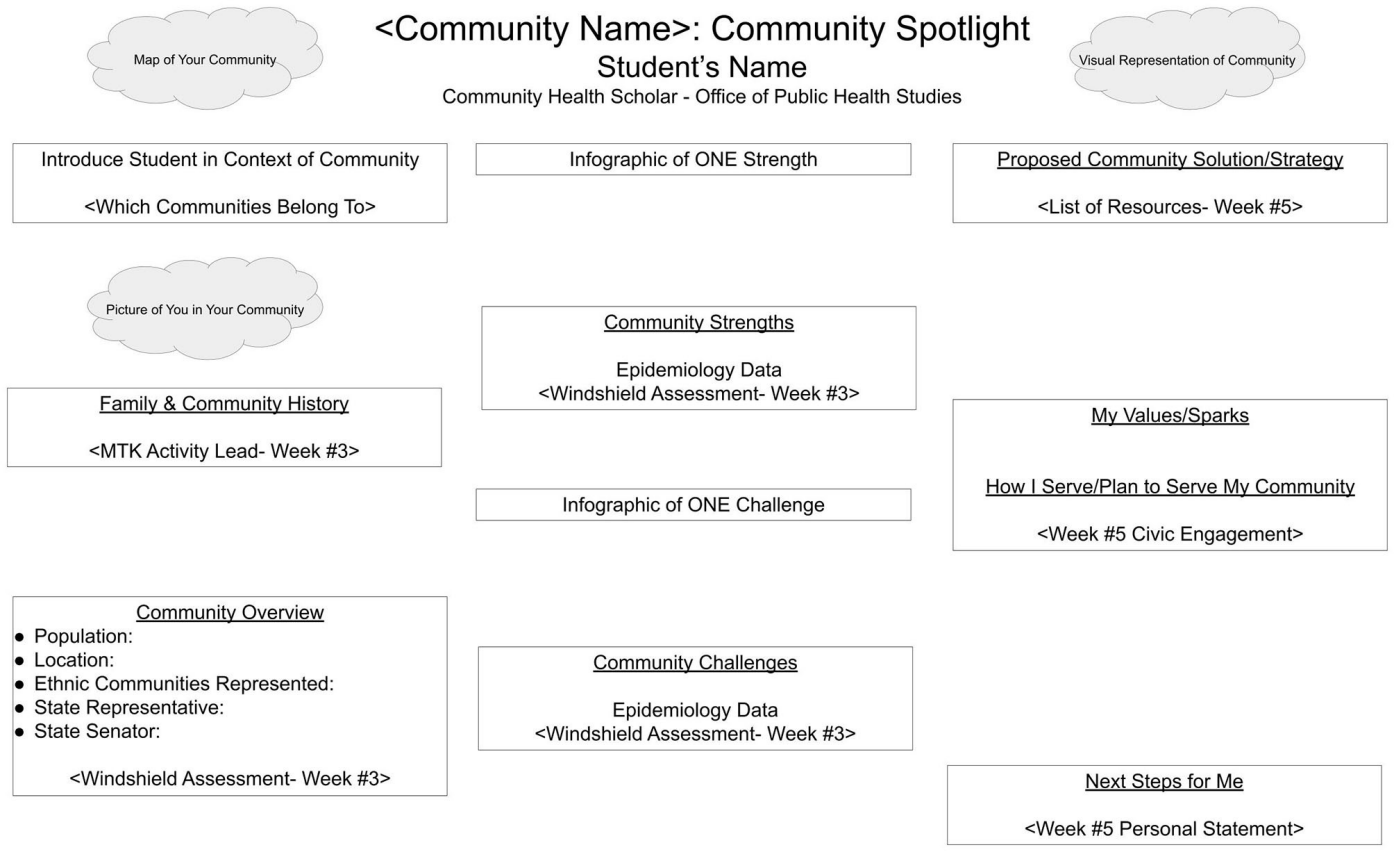
Sample student final product template.

Throughout the summer, students engaged weekly in activities integrating both their growing public health skills and knowledge with applications to better understand, and engage with, their local home communities. These scaffolded activities were conducted weekly (each Wednesday), and were shared as a culminating community highlight presented in a format similar to that of an academic poster (Figure 1). Students also engaged in personal development scaffolded throughout the summer program. Components of this developmental process included the following: (1) recognizing self in community, (2) conducting a community overview (windshield assessment and accessing existing data), (3) identifying community strengths and challenges, and finally (4) identifying community resources and opportunities for community engagement.

At the beginning of the program, students worked to identify the multiple diverse communities they were engaged with. Subsequent activities focused on their self-identified, geographic home community. Students applied recently acquired biostatistics and epidemiology knowledge and skills to seek out high-quality data describing the demographics of their community and interpret data and statistics to describe both strengths and challenges within their communities. Centered on discussions about environmental health, the built environment, and determinants of health, students developed, pilot tested, then applied a windshield assessment (45) in their home communities to collect qualitative data to complement publicly available community statistics. Complimented by discussions of health policy, students identified local state and county-level political representatives, and linked to subsequent discussions of behavioral change, identified local community organizations serving as community resources.

Final, student-developed community profile posters were shared at a public showcase and celebration following the conclusion of the program. In addition to student posters, a gallery walk display was also developed by students, with mentored support, to highlight activity work products, methods, and photos of activities students participated in throughout the program. Posters were given to students to take following the presentation; however, the gallery walk display was maintained for viewing by OPHS faculty, staff, and students for several months following program completion.

A summary of activities linked to identified Community Health Scholars learning objectives is provided in Table 2.

**Table 2:** Summer scholars learning objectives linked to program activities.

**Learning objectives**	**Program activities**
LEARN: Scholars complete PH 201—Introduction to Public Health alongside current college students	Foundational Coursework
ENGAGE: Scholars engage in interactive public health activities to supplement knowledge gained in the PH 201 course	Content Engagement Activities
GROW: Scholars engage in personal and professional development	Student Development Activities
PREPARE: Scholars assess, then build, college readiness skills	Student Development Activities
‘OHANA (Family): Participants have the opportunity to bring their family members to engage with events and workshops at specified intervals throughout the program to build familial support for college attendance and public health career pathways	‘*Ohana* (Family) Engagement Activities
COMMUNITY: Participants build connections with peers, near-peer college students, public health professionals, and members of the community	Foundational Coursework Student Development Activities Final Project and *Hō‘ike* (Showcase)

## 4 Results and assessment

### 4.1 Cohort overview

A total of eight students participated in our program in the summer of 2022. Three identified as male and five identified as female. Six were high school students at the time including, two juniors and four seniors. Two students were incoming 1st-year college freshmen. All students were from a local, public high school and seven of the eight students met the criteria of being from an ethnic background that is underrepresented in higher education at the University of Hawai'i at Mānoa.

### 4.2 Student scholar feedback

Throughout the summer program, students were provided with optional, weekly check-in surveys by email, to allow for opportunities for general feedback on the program as it progressed. Feedback was generally favorable, with opportunities identified for minor adjustments including the need to provide greater clarity in instructions and additional support in elements of the final project.

On the final day of the program, verbal feedback was collected from students in a loose focus group format facilitated by a public health faculty not previously affiliated with the program or known to the students. Students identified free college course credit as a primary motivation for participation. They were critical of the final project, expressing both technical challenges as well as low motivation, and suggested a more direct-to-community media engagement deliverable as an alternative. For example, a photovoice project or social media campaign about “What is Public Health,” to promote public awareness of the field of public health and its role in community health. Documentaries and public health films received mixed reviews, with some student appreciation but others who felt they were disengaging. Participation of guest speakers, both as lunch-time speakers and content presenters throughout the program, was not spoken of favorably. Students expressed wide variability, with many guest speakers focused on a lecture-based approach with minimal, if any, direct student engagement.

Interactive, public health-related engagement activities interwoven throughout the summer received positive feedback from students, however, students expressed interest in additional activities related to social-emotional learning, as well as a desire for more workshops related to the development of college success skills. Overall, students expressed feeling cared about throughout the summer program and particularly appreciated encouragement by program faculty to continue engagement.

### 4.3 Program faculty debrief findings

Feedback was also collected from debrief meetings of the two program faculty- the program coordinator, and the course instructor, both of whom were involved in the full program schedule. Data were compiled and summarized by program faculty from notes taken during debrief discussions on the final day of the program, 2 weeks following program completion, and roughly 6 months later. The length of the program day, 9 am−2:30 pm, was identified as too long. Students rarely needed more than 15–20 min as a lunch break and reported getting bored and tired during the long lunch hour. Program faculty, too, felt several activities may be condensed in time frame, and lack of student enthusiasm with both guest speaker engagements and documentary viewing-discussion sessions, may yield opportunities to achieve similar program outcomes in a shorter time frame. Pandemic-associated grant funding of student lunches also created concerns regarding the sustainability of providing a meal.

Throughout the program, college success skill activities were conducted primarily by guest speakers from campus and community partner organizations, rather than program faculty. While the intent was to showcase a diversity of resource contacts, a greater focus on a consistent pedagogical approach seemed to be more valuable and effective. Critical feedback from students regarding guest speakers overall may be associated with the increased demand for college success skill activities, especially if shared by program faculty or partners with a similar active learning approach. Another area for further development included the need to promote communication skills among student scholars. Often students found themselves challenged with articulating their thoughts and understanding to share with peers, and to a greater extent, with program faculty and community professionals. Greater scaffolded support and training should be devoted to this skill area in the future.

## 5 Discussion and lessons learned

Plans are currently in place to offer the Community Health Scholars Program in the summer of 2023. Moving forward, program faculty have made adjustments to strategies to recruit prospective students, including more targeted outreach to local high school counselors and specifically to teachers at high schools that offer students either a health academy program or public health-related coursework. We hope these adjustments to recruitment will help to expand interest and increase the number of applicants, and subsequent participants. Thus, far, 12 students have applied for summer 2023, 11 have met program inclusion criteria, and have been invited to participate. Seven students have committed to program participation. A modified schedule will include repeating the introduction to public health course from 9 am to 10:15 am but will include two subsequent 50-min program sessions, allowing for program dismissal at 12:30 pm, rather than 2:30 pm. Shortening the program time can also provide students the opportunity to have a summer job, participate in other summer activities, or fulfill family obligations.

Based on findings and feedback, revisions to future program implementation will include similar course-based and content-engagement activities. However, student development activities will be expanded to include scaffolded support and training in verbal communication and other forms of expression, including encouragement of active, respectful engagement with both peers and professionals. Integration of guest speakers will be more judicious, with active encouragement and support by program faculty to incorporate active learning strategies during student engagement.

Constructive feedback from both students and faculty regarding the final project suggested a reimagining of the scaffolded, community-specific program activities and culminating product. Current plans center around community-specific program activities to be modified and shared as a visual portfolio collection of work products to be displayed during the program's *Hō‘ike* event. To address interest in a media-forward product, the cohort of students will be given the opportunity to decide how they together would like to showcase their summer experiences for their families and community guests. Suggestions from program faculty include the development of a brief summary video or slideshow.

Looking to the future, there are concerns regarding the sustainability of funding to support both student tuition costs and personnel expenses, as well as financially supporting student transportation costs to access the University of Hawai'i at Mānoa campus. While one of the biggest motivators to participate in the program was free college credit, we recognize that students from underrepresented and marginalized populations in higher education may need additional financial support (e.g., a stipend or available time for employment), while participating in summer learning experiences. This project was initiated as part of a temporary extramural funding opportunity. The summer time commitment is also substantial for faculty, especially those who also engage heavily in student support and instruction during the academic year. There are additional concerns regarding the return, in terms of student applicants, on faculty engagement in outreach and recruitment efforts to local high schools and college counselors, especially as student participation continues to be lower than anticipated. However, in examining early long-term outcomes from scholars a year following the initial 2022 cohort, all five eligible participants are pursuing higher education, and of them, two scholars are either enrolled or enrolling in bachelor's level public health degree programs. The remaining three scholars are current high school students. While financial and time costs are meaningful, the benefits and return on investment in terms of increased student enrollment and diversity are also substantial, with long-term, compounding impacts and implications in increased access to higher education and subsequently to the public health workforce. This program is also easily scalable to accommodate larger enrollment in the range of 50–75 students per cohort, with additional, incremental costs almost entirely focused on per-student enrollment fees (roughly 260 USD).

It is our hope that through initiatives such as the Community Health Scholars Program, the academic pipeline, and subsequent public health workforce will increase in diversity to be increasingly reflective and representative of the communities they serve.

## Data availability statement

The raw data supporting the conclusions of this article will be made available by the authors, without undue reservation.

## Ethics statement

The requirement of ethical approval was waived by University of Hawai'i Institutional Review Board, Human Studies Program, University of Hawai'i for the studies involving humans because identified as not human subjects research. The studies were conducted in accordance with the local legislation and institutional requirements. Written informed consent for participation in this study was provided by the participants' legal guardians/next of kin.

## Author contributions

DN-H: Formal analysis, Funding acquisition, Methodology, Project administration, Resources, Supervision, Validation, Visualization, Writing – original draft, Writing – review & editing. MT-K: Conceptualization, Formal analysis, Funding acquisition, Methodology, Project administration, Resources, Supervision, Validation, Visualization, Writing – original draft, Writing – review & editing.
